# Hydrothermal Pretreatment of Date Palm (*Phoenix dactylifera* L.) Leaflets and Rachis to Enhance Enzymatic Digestibility and Bioethanol Potential

**DOI:** 10.1155/2015/216454

**Published:** 2015-08-12

**Authors:** Chuanji Fang, Jens Ejbye Schmidt, Iwona Cybulska, Grzegorz P. Brudecki, Christian Grundahl Frankær, Mette Hedegaard Thomsen

**Affiliations:** ^1^Institute Center for Energy (iEnergy), Masdar Institute of Science and Technology, P.O. Box 54224, Abu Dhabi, UAE; ^2^Department of Chemistry, Technical University of Denmark, 2800 Kongens Lyngby, Denmark

## Abstract

Date palm residues are one of the most promising lignocellulosic biomass for bioethanol production in the Middle East. In this study, leaflets and rachis were subjected to hydrothermal pretreatment to overcome the recalcitrance of the biomass for enzymatic conversion. Evident morphological, structural, and chemical changes were observed by scanning electron microscopy, X-ray diffraction, and infrared spectroscopy after pretreatment. High glucan (>90% for both leaflets and rachis) and xylan (>75% for leaflets and >79% for rachis) recovery were achieved. Under the optimal condition of hydrothermal pretreatment (210°C/10 min) highly digestible (glucan convertibility, 100% to leaflets, 78% to rachis) and fermentable (ethanol yield, 96% to leaflets, 80% to rachis) solid fractions were obtained. Fermentability test of the liquid fractions proved that no considerable inhibitors to *Saccharomyces cerevisiae* were produced in hydrothermal pretreatment. Given the high sugar recovery, enzymatic digestibility, and ethanol yield, production of bioethanol by hydrothermal pretreatment could be a promising way of valorization of date palm residues in this region.

## 1. Introduction

High worldwide demand for energy, unstable and uncertain petroleum sources, and concern over global climate change have led to a resurgence in the development of alternative energy that can displace fossil transportation fuel [1]. The idea of converting biomass-derived sugars to transportation biofuels was first proposed in the 1970s. Once again, the idea is being seriously contemplated as a possible substitute for petroleum-based liquid fuels. Economic and geopolitical factors (high oil prices, environmental concerns, and supply instability) have certainly played a role in reviving interest in renewable resources [2]. Evidence suggests that transportation fuels based on lignocellulosic biomass represent the most scalable alternative fuel source. Lignocellulosic biomass in the form of plant materials (e.g., grass, wood, and crop residues) offers the possibility of a renewable, geographically distributed, and greenhouse-gas-favorable source of sugars that can be converted to ethanol and other liquid fuels [3]. Geopolitical factors have strongly driven the research, as has commercialization of cellulosic biofuels in the past 7 years in North and South America, Europe, and China. The US Department of Energy (DOE) in 2008 established five research centers at a total cost of more than $300 million. In addition, in 2009 DOE committed $480 million to improve the energy efficiency of biofuels and biomass conversion plants. In the European Union (EU), the total contribution of biofuel projects (mostly second generation) under the Seventh Framework Program adds up to €45 million [4]. The first commercial-scale cellulosic ethanol refinery (owned by Beta Renewables, Italy) opened in Italy in 2013 [5]. DuPont Biofuel Solutions, a subsidiary of DuPont, is constructing a commercial-scale cellulosic ethanol facility built for an estimated $276 million with a capacity of 25 MGY of ethanol near Nevada, IA. POET and DSM are jointly constructing a 20 MGY cellulosic ethanol plant in Emmetsburg, IA [6]. In China, there were eight pilot and demonstration plants in operation by December 2009, with a total capacity of 280,500 tons bioethanol per year [7]. Due to the typical geography and climate of the Middle East, researches have been focusing on using marine (e.g., macroalgae) biomass [8], halophytes (e.g.,* Salicornia bigelovii*) [9], and agricultural wastes (e.g., date palm fruit and sap) [10, 11] to produce bioethanol.

The date palm (*Phoenix dactylifera* L.) is a major fruit crop in most Middle Eastern countries. It has historically been connected to sustaining human life and traditional heritage of the people in the old world as a major agricultural crop. Middle Eastern countries possess 70% of the 120 million world's date palms [12]. Generally, each date palm tree produces 10 to 30 dried leaves annually. An average naturally dried leaf (includes leaflets and rachis) has a mass of 2-3 kg [13]. Hence, each date palm is estimated to yield approximately 50 kg leaf residues per year. This means the annual yield of lignocellulosic feedstock from date palm leaf residues is over 4 million tons. Regardless of the abundant lignocellulosic biomass, to our knowledge, no study has been conducted on bioethanol production from lignocellulosic date palm residues.

Plant biomass has evolved complex structural and chemical mechanisms for resisting assault on its structural sugars from predators such as microbes, insects, and animals contributing to the recalcitrance of lignocellulosic feedstock to chemical or enzymatic conversion. A pretreatment step is usually conducted to reduce recalcitrance by depolymerizing and solubilizing hemicellulose. Removal of hemicellulose from the microfibrils is thought to expose the crystalline cellulose core, which can then be hydrolyzed by cellulolytic enzymes [1]. Hydrothermal pretreatment (also known as autohydrolysis or liquid hot water pretreatment) has attracted a great deal of attention because it can be considered as an eco-friendly green processing technology by using water and steam only. One of the most important benefits of using water instead of acid as pretreatment media is that it avoids corrosion problems, acid recycling, and the formation of neutralization sludge. Another advantage is that hydrothermal pretreatment tends to result in lower inhibiting hydrolyzates which can decrease yield in the subsequent fermentation process [14]. Petersen et al. [15] reported an optimum hydrothermal pretreatment condition of wheat straw to be 195°C for 6–12 min, which yielded 70% hemicellulose recovery and 93-94% cellulose recovery in the fibers, and approximately 89% of the cellulose was converted into ethanol by commercial cellulase mixture. Kumar et al. [16] showed that almost 100% cellulose and 92% hemicellulose were recovered from hydrothermal pretreated switch grass, and more than 80% of glucan digestibility was achieved at 190°C for 20 min. In the study of Romaní et al. [17] up to 94% of polysaccharides were recovered in the hydrolysis media as mono- or oligosaccharides when using* Eucalyptus globulus* wood pretreated at 220°C.

Despite the wide interest in biomass for energy production scaling up experimental projects to commercial operations is not easy [18]. Estimation of energy (e.g., bioethanol) production potential is a necessary starting point to reveal the specific dynamics and interrelationships between environmental and socioeconomic systems [19]. Wild-type* S. cerevisiae* strains readily ferment C6 sugars including glucose, mannose, fructose, and galactose as well as the disaccharides sucrose and maltose [20]. On the other hand, other of the most abundant sugar monomers from biomass D-xylose and L-arabinose (C5 sugars) require either extensive metabolic engineering of* S. cerevisiae* [20] or other fermentative organisms such as* Kluyveromyces marxianus* [21],* Zymomonas mobilis* [22], and* Pichia stipitis* [21].

The primary focus of this research is to evaluate lignocellulosic biomass of date palm (leaflets and rachis) as potential bioethanol feedstock. Hydrothermal treatment was chosen as a pretreatment method of overcoming biomass recalcitrance prior to enzymatic hydrolysis and fermentation in order to facilitate high yield in both processes. Morphological and chemical changes by pretreatment were observed by scanning electron microscopy (SEM) and attenuated total reflection-Fourier transform infrared spectroscopy (ATR-FTIR). X-ray diffraction (XRD) was used to compare the crystallinity of date palm leaflets and rachis before and after hydrothermal pretreatment.

## 2. Materials and Methods

### 2.1. Materials and Preparation

Leaves were collected from date palm trees in Abu Dhabi in 2013. Leaflets and rachis were separated from leaves. They were dried and stored before use. The dried material was milled using a knife mill (IKA, 10 MF Basic) to pass through a 1 mm screen.

### 2.2. Biomass Chemical Composition Analysis

Sequential Soxhlet extractions with water and ethanol were performed based on National Renewable Energy Laboratory (NREL) protocol [23]. Structural carbohydrates and lignin of extractives-free date palm leaflets and rachis before and after hydrothermal pretreatment were subjected to two-step acid hydrolysis according to the analytical procedure of NREL [24]. The hydrolyzates were analyzed for sugars using High Performance Liquid Chromatography (Agilent 1260 Infinity Bio-Inert Binary LC). The Hi Plex-H column (Agilent) and refractive index detector (RID) were used to determine the concentrations of glucose, xylose, and arabinose at 65°C using 0.005 M H_2_SO_4_ as the mobile phase (eluent) with a flow rate of 0.6 mL/min.

### 2.3. Hydrothermal Pretreatment

Hydrothermal pretreatment experiments were performed at 10% w/w dry matter loading, at four temperature levels (180, 190, 200, and 210°C). Processing time was maintained at 10 min. Combined severity factors corresponding to each condition were calculated [25]

(1) $$\begin{array}{c} \text{CS}\left(\text{combined severity}\right)=\log\left(R_{o}\right)-\text{pH}=\log\left(t\exp\left(\frac{T-100}{14.75}\right)\right)-\text{pH}, \end{array}$$

where *T* is temperature, °C; *t* is time, min; *R*_*o*_ is severity factor.

The pretreatment was conducted in a Parr reactor (Parr Instrument Company, Moline, Illinois) with working volume of 1 liter. For all experiment, it took less than 15 minutes to reach the targeted temperature, and the relaxation time was short (<10 min). After the treatment, the reactor was cooled to 40°C and the pretreated material was separated by filtration into solid (fibers) and liquid fraction (filtrate). Both fractions were kept at 4°C until analysis and further processing. Further processing included the following: composition analysis of the fiber and liquid fractions, enzymatic hydrolysis of fibers, convertibility of the fibers in SSF with* S. cerevisiae*, and fermentability by* S. cerevisiae* of the liquid fraction. Mass balance of all the crucial components (glucan and xylan) was performed using (2)–(4). Content of these components in the solid fractions was measured using two-step acid hydrolysis [24], while their amount in the liquid fractions (filtrates) was measured using dilute-acid hydrolysis described later in this section:

(2) $$\begin{array}{c} \text{Dry Mass in}\left(\text{g}\right)=\frac{{\text{TS}}_{\text{raw}}*W_{\text{initial biomass}}*C_{\text{raw}}}{100*100}, \end{array}$$

where *W*_initial biomass_ is weight of the initial biomass fed into the pretreatment [g]; TS_raw_ are total solids in the raw biomass [%]; *C*_raw_ is content of the specific component in the raw biomass measured by strong acid hydrolysis [g/100 g TS]. Thus,

(3) $$\begin{array}{c} \text{Dry Mass out}\left(\text{g}\right)=\frac{{\text{TS}}_{\text{pretreated}}*W_{\text{fibers}}*C_{\text{fibers}}}{100*100}, \end{array}$$

where *W*_fibers_ is weight of the biomass in fiber fraction after the pretreatment [g]; TS_pretreated_ are total solids of the pretreated biomass [%]; *C*_fibers_ is content of the specific component in the fiber fraction after the pretreatment measured by strong acid hydrolysis [g/100 g TS]. Hence,

(4) $$\begin{array}{c} \text{Fiber fraction recovery}\;\%=\frac{\text{Dry mass out}\left(\text{g}\right)}{\text{Dry mass in}\left(\text{g}\right)}*100\%,\\ \text{Liquid fraction recovery}\;\%=\frac{\text{Amount in the filtrate}\left(\text{g}\right)}{\text{Dry mass in}\left(\text{g}\right)}*100\%. \end{array}$$

### 2.4. Fiber Fraction Analysis and Further Processing

#### 2.4.1. Fiber Composition Analysis

The pretreated fibers were subjected to two-step acid hydrolysis [24] to determine sugar recovery. The process was carried out following the same protocol as in the case of the extractives-free raw leaflets and rachis described above in Section 2.2.

#### 2.4.2. Enzymatic Hydrolysis

Enzymatic convertibility assay based on commercial Cellic CTec2 (117 FPU/mL, protein content 194 mg protein/mL) and Cellic HTec2 enzymes (Novozymes A/S, Denmark) was used to determine the efficiency of the pretreatment. Protein concentration of enzymes was determined as described by Bradford [26]. The hydrolysis was performed according to National Renewable Energy Laboratory protocol [27] using 100 g/L dry biomass loading and 15 FPU cellulase/g dry matter of biomass (with cellulase-to-hemicellulase ratio of 1 : 9). The process was performed in the presence of 50 mM citrate buffer (pH 5) at 50°C and samples were shaken at 150 rpm for 72 h. Glucose released during the enzymatic hydrolysis was quantified using HPLC at the same operating conditions as applied in the acid hydrolysis samples (described above). Enzymatic convertibilities of cellulose to glucose and xylan to xylose were calculated using

(5) $$\begin{array}{c} \text{Convertibility}\left(\%\right)=\frac{C_{\text{glucose/xylose}}}{L*\left(C_{\text{fibers}}/100\;\text{g}\;\;\text{TS}\right)}*100\%, \end{array}$$

where *C*_glucose/xylose_ is concentration of glucose/xylose measured in the enzymatic hydrolyzate [g/L]; *L* is fibers loading [g/L]; *C*_fibers_ is content of the specific component (glucose or xylose) in the fiber fraction after the pretreatment [g/100 g TS].

Based on the glucose and xylose yield by enzymatic hydrolysis, bioethanol potential was calculated using

(6) $$\begin{array}{c} \text{Bioethnaol potential}\left(\frac{\text{kg}}{t\;\;\text{dry biomass}}\right)=1000*C_{\text{glu/xyl}}*\frac{1}{100}*R_{\text{glu/xyl}}*f_{\text{carbohydrate-to-monosugar}}*Y_{\text{glu/xyl}}*0.511, \end{array}$$

where *C*_glu/xyl_ is glucan/xylan content in raw biomass [g/100 g TS]; *R*_glu/xyl_ is glucose/xylose recovery; *f*_carbohydrate-to-monosugar_ is glucan-to-glucose factor (1.11), xylan-to-xylose factor (1.14); *Y*_glu/xyl_ is glucose/xylose conversion by enzymatic hydrolysis; 0.511 is glucose/xylose-to-ethanol factor [19].

#### 2.4.3. Simultaneous Saccharification and Fermentation (SSF)

The fermentation mixture was prepared in 250 mL flasks using 100 mL total volume. The flasks were secured with yeast locks to ensure anaerobic conditions while enabling carbon dioxide release. The mixture was composed of enzymes (types and doses the same as for enzymatic hydrolysis), pH buffer (to maintain the pH at 5.0), and 10% DM (dry mass) biomass loading. The mixture was prehydrolyzed for 24 hours at 50°C and 150 rpm in a shaking bed incubator. After the prehydrolysis the solution was cooled to 32°C and 0.2 g of dry Baker's yeast (*S. cerevisiae*) was added along with urea as nutrients source (0.2 mL of a 24% urea solution). The process was performed at 32°C with constant shaking (90 rpm) for 72 hours. Weight measurements of the flasks were recorded throughout the duration of the process. The weight loss (representing release of carbon dioxide) was translated to ethanol yield using (7). Final ethanol concentration was determined by the HPLC analysis:

(7) $$\begin{array}{c} W_{\text{ethanol}}=\frac{1\;\text{mol}\;\;\text{EtOH}}{1\;\text{mol}\;\;{\text{CO}}_{2}}*\frac{M_{\text{ethanol}}}{M_{{\text{CO}}_{2}}}*W_{{\text{CO}}_{2}}, \end{array}$$

where *W*_ethanol_ is weight of ethanol (EtOH) produced [g]; *M*_ethanol_ is molar mass of ethanol [46 g/mol]; *M*_CO_2__ is molar mass of CO_2_ [44 g/mol]; *W*_CO_2__ is weight of CO_2_ produced = weight loss of the fermentation flask [g].

Theoretical ethanol yield was determined based on the glucan content in the raw material following (8). Ethanol yield (%) was calculated as a percent ratio of the actual ethanol amount produced to the theoretical ethanol yield (9):

(8) $$\begin{array}{c} {\text{TY}}_{\text{ethanol}}=0.511*C_{\text{glucan}}*L*1.11, \end{array}$$

where TY_ethanol_ is theoretical ethanol yield [g]; *C*_glucan_ is glucan content in fibers; *L* is fibers loading [g/L]; 0.511 is glucose-to-ethanol factor; 1.11 is glucan-to-glucose factor. Hence,

(9) $$\begin{array}{c} Y_{\text{ethanol}}=\frac{\text{Ethanol}\;\;\text{produced}\left(\text{g}\right)}{{\text{TY}}_{\text{ethanol}}\left(\text{g}\right)}*100\%. \end{array}$$

### 2.5. Liquid Fractions (Filtrates) Analysis

#### 2.5.1. Analysis of Free Sugars and Pretreatment By-Products

Liquid fractions were analyzed for the released (free) monomeric sugars including glucose, xylose, and arabinose and for the pretreatment by-products (mostly sugar degradation products) including acetic acid, furfural, and HMF. The analysis was performed using HPLC with the same operating conditions as described above.

#### 2.5.2. Dilute-Acid Hydrolysis

To determine the quantity of the oligomers of xylose, arabinose, and glucose in the pretreated liquid fraction (filtrate) dilute-acid hydrolysis was carried out with 8% w/w H_2_SO_4_ solution according to Cybulska et al. [9]. The ratio of the sample to acid was 1 : 1, producing a final acid concentration of 4% w/w. The solution was autoclaved at 121°C for 10 min. Sugar recovery measurement was performed and the recovery factor was included in the calculations to account for sugar losses to degradation. The hydrolyzates were analyzed using the HPLC system to measure the concentrations of the simple sugars released. The results were then used in the mass balance recovery calculations for the sugars, as described above.

#### 2.5.3. Fermentability Study

Fermentability study was done according to Cybulska et al. [9]. The liquid fractions obtained from the pretreatment process were fermented in order to test the possibility of using the filtrates as fermentation medium. Since the liquid fraction contains fewer hexoses (as compared to solid fractions) and more inhibitors, glucose (35 g/L) was added to the fermentation solution. Flasks containing hydrolysates, glucose, and 2 g/L of* S. cerevisiae *were incubated at 32°C and 100 rpm. Weight loss measurements were taken once a day for 72 hours, and the weight loss was converted to ethanol yield using (9).

### 2.6. Scanning Electron Microscopy (SEM)

A Quanta 250 (FEI, Oregon, USA) scanning electron microscopy operated at 2 keV was used to image leaflets and rachis before and after pretreatment. Samples were coated with gold using a vacuum sputter-coater to improve the conductivity of the samples and thus the quality of the SEM images.

### 2.7. Attenuated Total Reflection-Fourier Transform Infrared Spectroscopy (ATR-FTIR)

Attenuated total reflection-Fourier transform infrared spectroscopy (ATR-FTIR) was conducted using a Bruker Optics Vertex system (Bruker, Massachusetts, USA) with built-in diamond–germanium ATR single reflection crystal. Leaflets and rachis before and after pretreatment were pressed uniformly against the diamond surface using a spring loaded anvil. Sample spectra were obtained using an average of 64 scans over the range of 500 and 4,000 cm^−1^ with a spectral resolution of 4 cm^−1^.

### 2.8. X-Ray Diffraction (XRD)

Dewaxing [28] was deployed in leaflets (with and without pretreatment) to avoid the crystallographic interference by wax. Rachis (with and without pretreatment) and dewaxed leaflets were analyzed by Empyrean X-ray diffractometer (PANalytical, Eindhoven, Netherlands) equipped with a PIXcel3D detector and operated at 45 kV and 40 kA using Cu K*α* radiation (*λ* = 1.5418 Å). Powder diffraction data were collected in reflection geometry in the 2*θ* range of 10 to 40° with a step size of 0.008° and a counting time of 10 s per step.

The Rietveld method was adopted to estimate the percent crystallinity in the biomass samples as this method takes into account the overlapping and widely broadened diffraction peaks of cellulose [29]. Refinements were performed as described by Thygesen et al. [29] using the crystal structure of cellulose I*β* as input. The peak shapes were modeled by the Voigt function, and a total of 15 parameters were refined to find the best fit for all samples. The best parameter set included refinement of one scale factor, 10 Chebyshev background parameters, one parameter accounting for the sample transparency effect, one Voigt peak profile parameter, the unit cell parameter *b*, and one preferred orientation parameter along (130). Other unit cell parameters were fixed to those reported by de Figueiredo and Ferreira [30].

## 3. Results and Discussions

### 3.1. Composition Analysis and Sugar Recovery

**Figure 1:**
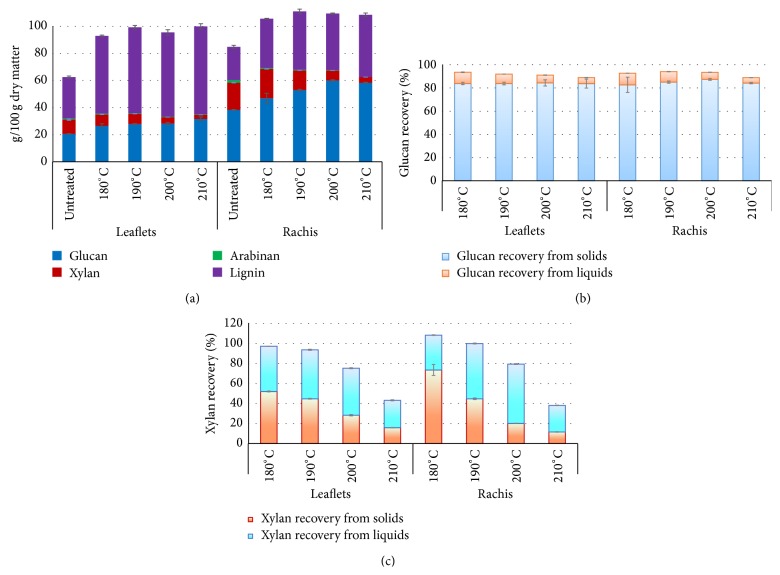
Main chemical components and sugar recovery of leaflets and rachis of before and after hydrothermal pretreatment. (a) Main chemical components (glucan, xylan, arabinan, and lignin) analysis. (b) Glucan recovery from solids and liquids of pretreated leaflets and rachis. (c) Xylan recovery from solids and liquids of pretreated leaflets and rachis.

The main chemical components (glucan, xylan, arabinan, and lignin) of leaflets and rachis before and after pretreatment are shown in Figure 1(a). Glucan and xylan content was 20.62% and 10.53% (based on dry matter) in leaflets, while it was 38.34% and 20.07% in rachis. Lignin content in leaflets and rachis was 30.54% and 24.61, respectively. The remaining components were mainly extractives and ashes. Solids recovery for all pretreated biomass was more than 97% (data not shown). Both glucan and lignin content increased (percent basis) significantly after pretreatment. The highest glucan and lignin content was 31.63% and 64.95% in pretreated leaflets at 210°C and was 60.12% for glucan at 200°C and was 45.95% for lignin at 210°C in pretreated rachis. Xylan contents in pretreated leaflets and rachis both decreased, with the lowest value observed at 210°C (3.07% for leaflets and 4.22% for rachis).

High sugars recoveries were obtained by hydrothermal pretreatment of leaflets and rachis (Figures 1(b) and 1(c)). Glucan recoveries of both pretreated leaflets and rachis were above 90%. More than 75% and 79% xylan was recovered from pretreated leaflets and rachis, respectively, except those pretreated at 210°C. There was a dramatic drop of xylan recovery (less than 50%) for either biomass when the temperature was higher than 200°C. In solids, more than 80% of glucan was recovered in pretreated leaflets and rachis. While only less than 10% glucan was recovered from liquids. By contrast, maximum xylan recovery was 52% and 74% in solids of pretreated leaflets and rachis. Relatively low xylan recoveries in solid fractions were results of leaching of xylan to liquid fractions (27%–49% for leaflets and 27%–74% for rachis).

Composition analysis (Figure 1(a)) shows that carbohydrate contents (glucan and xylan) of rachis are comparably high with conventional lignocellulosic biomass around the world like corn stover (30%–38% and 20%–25%), wheat straw (34%–40% and 21%–26%), and sugarcane bagasse (32%–43% and 22%–25%) [31]. Moreover, rachis has slightly lower lignin content than other woody biomass like eucalyptus (29–32%) and pine (28%) [32]. Given the high carbohydrates and low lignin content, date palm rachis seems to be a potential source of woody biomass for biorefinery. Conversely, leaflets have significantly lower carbohydrates content and higher lignin content than conventional lignocellulosic biomass, making date palm leaflets a potential biomass candidate for lignin production. The obvious increases of glucan and lignin content in the pretreated biomass are mainly due to the removal of xylan whose degradation is more sensitive to temperature. The results in Figures 1(b) and 1(c) show that hydrothermal pretreatment is capable of achieving high glucan (higher than 90%) and xylan (higher than 75%) recovery at moderate pretreatment conditions (180–200°C) without adding any chemicals, which is agreeing with the work done on other conventional lignocellulosic biomass including wheat straw, switchgrass, and* Eucalyptus globulus* wood [14–16]. Combined severity factor is often used to describe the removal of lignin and solubilization of xylan. There is a good correlation between the combined severity factor and xylan recovery of pretreated leaflets (*R* ^2^ = 0.93) and rachis (*R* ^2^ = 0.89), showing the potential of using combined severity factor as an index of xylan recovery in pretreatment process optimization. Similar trend was observed in other studies on hydrothermal pretreatment of* S. bigelovii* straw [9] and dilute sulfuric acid pretreatment of corn stover [33].

### 3.2. Effect of Hydrothermal Pretreatment on Morphological Changes

**Figure 2:**
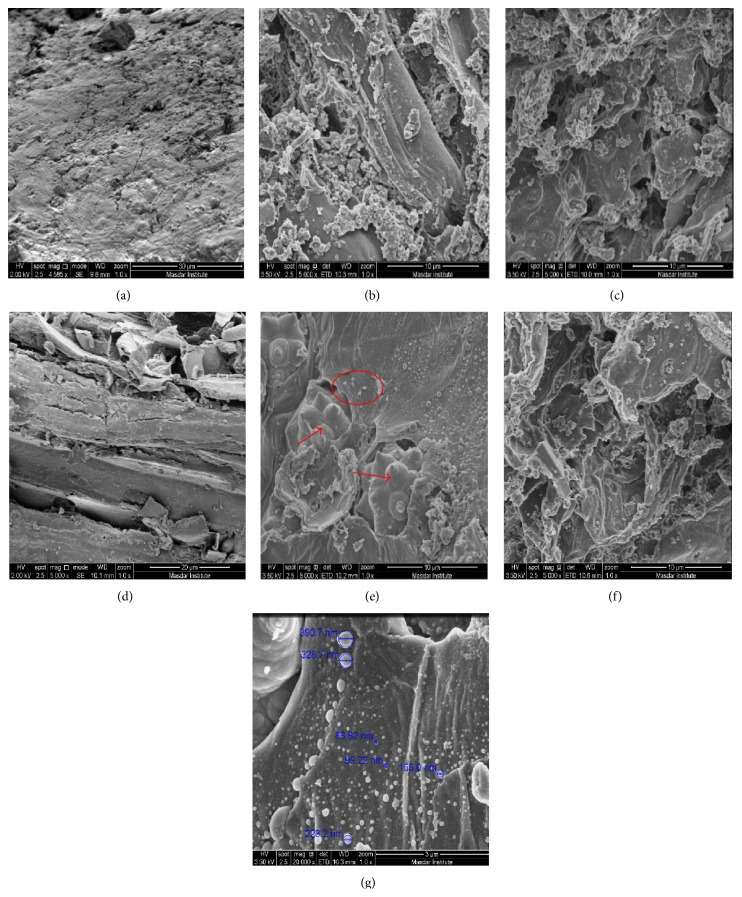
Scanning electron micrographs of untreated and pretreated date palm leaflets and rachis. (a) Raw leaflets. (b) and (c) Leaflets treated at 180°C and 210°C, respectively. (d) Raw rachis. (e) and (f) Rachis treated at 180°C and 210°C, respectively. (g) Magnified red circle area in (e). Red arrows represent crystallites in (e).

The surface morphologies of raw (before pretreatment) leaflets and rachis and pretreated ones are shown in Figures 2(a)–2(f).  Figure 2(g) is the magnified image of the area labelled in red circle on Figure 2(e). Surface of rachis (Figure 2(d)) was more irregular than that of leaflets (Figure 2(a)). A few bundles of fibers were observed on the surface of rachis. In addition, there was evident morphological contrast of leaflets and rachis before and after pretreatment according to Figures 2(a)–2(f). Structure of biomass after pretreatment was greatly changed, forming a variety of smaller fragments. The changes caused by pretreatment at 210°C were more significant than those by treatment at 180°C for both leaflets and rachis. Spherical droplets with smooth surface texture (less than 1 *μ*m in diameter) scattering on the surface were noticed after pretreatment as shown in Figures 2(e) and 2(g). Figure 2(e) (rachis pretreated at 180°C) showed some crystallites half buried, which were not observed in Figure 2(f) (rachis pretreated at 210°C).

Hydrothermal pretreatment caused significant morphological changes to both leaflets and rachis, showing the effectiveness of the pretreatment. Generally, higher pretreatment temperature caused more destruction of cell wall structure of both leaflets and rachis. Consequently, the destruction of cell wall increases the surface areas that are accessible to cellulolytic enzymes, which enhances cellulose enzymatic convertibility of lignocellulosic biomass. Donohoe et al. [34] characterized the resulting surface features of pretreated corn stover by several techniques, such as FTIR, NMR (nuclear magnetic resonance) analysis, antibody labeling, and cytochemical staining. It was hypothesized that thermochemical pretreatments reaching temperatures above the range of lignin phase transition caused lignin to coalesce into larger molten bodies that migrate within and out of the cell wall and can redeposit on the surface of plant cell walls. In our observations, the droplets exhibited similar physical features described in [34–36] based on their shape (spherical), size (up to 1 *μ*m), and surface texture (smooth exterior). Another hypothesis for the spherical droplets is that they are pseudolignin derived from carbohydrate like xylan during severe hydrothermal pretreatment [37, 38]. Lignin and lignin-like material (pseudolignin) was hypothesized to be responsible for the spherical droplets formation as visualized by SEM in our study. It was reported in other studies that the spherical droplets formation is possibly responsible for lower enzyme efficiency during enzymatic hydrolysis of pretreated materials, which was suspected to be caused by reduced accessibility of enzymes to substrates [36, 39]. The crystallites found in Figure 2(e) (labeled by red arrows) are probably the minerals exuding from fibers and vessels. The presence of these crystal minerals confirms the conductive function of the fibers and vessels [40].

### 3.3. Effect of Hydrothermal Pretreatment on Chemical and Structural Changes

**Figure 3:**
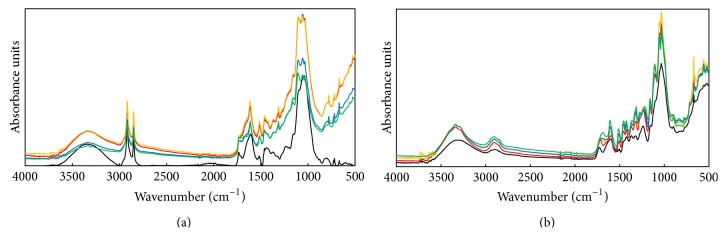
Attenuated total reflection-Fourier transform infrared spectroscopy (ATR-FTIR) of date palm leaflets and rachis. (a) FTIR spectra of the date palm leaflets in the range of 3,500 to 500 cm^−1^. The black line represents the leaflets. The red line, yellow line, blue line, and green line represent the pretreated leaflets at 180°C, 190°C, 200°C, and 210°C, respectively. (b) FTIR spectra of the date palm rachis in the range of 3,500 to 500 cm^−1^. The black line represents the rachis. The red line, yellow line, blue line, and green line represent the pretreated leaflets at 180°C, 190°C, 200°C, and 210°C, respectively.

Tracking the chemical and structure changes of cellulose, hemicellulose, and lignin by FTIR facilitates mechanical study of the effects of pretreatment on the enzymatic digestibility of biomass.  Table 1 describes the FTIR absorbance bands used to monitor the chemical and structure changes of lignocellulosic biomass. Figures 3(a) and 3(b) show the spectroscopy of date palm leaflets and rachis in the range of 3,500 to 500 cm^−1^.

The small band at 900 cm^−1^ representing amorphous cellulose was observed in rachis while it was hardly found in leaflets, showing one of the differences of crystallinity between leaflets and rachis. Moreover, rachis samples showed increase of amorphous cellulose peak intensity when increasing the pretreatment temperature from 180°C to 210°C. It indicated that more amorphous cellulose was achieved by hydrothermal pretreatment with increased pretreatment severity. Another band around 1,098 cm^−1^ responsible for crystalline cellulose was observed in both leaflets and rachis. Stronger peaks at around 1,098 cm^−1^ were generated after pretreatment of both leaflets and rachis, suggesting an increasing proportion of crystalline cellulose in pretreated biomass due to the removal of amorphous cellulose compared with raw biomass. For leaflets, the wide band at around 1,035 cm^−1^ diverged into two small peaks at 1,035 cm^−1^ and 1,045 cm^−1^ after pretreatment. Similar change of band at around 1,035 cm^−1^ was observed between raw rachis and pretreated ones. These bands can be assigned to different bonds vibration coming from lignin, cellulose, and hemicellulose (Table 1). The region around 1,247 cm^−1^ was characterized to represent hemicellulose and lignin disappears after pretreatment of leaflets. Pretreated leaflets and rachis exhibited stronger peaks than untreated ones at three peaks (1,465, 1510, and 1,595 cm^−1^) that are responsible for lignin.

The pretreatment increases the crystallinity of cellulose for both leaflets and rachis. Only for rachis the decrease of crystalline cellulose was observed when raising temperature from 180°C to 210°C. It is consistent with the crystallinity profiles shown in Table 2. The signal in region between 1,200 and 1,000 cm^−1^ is a large contribution of hemicellulose and cellulose, which exhibits a maximum value around 1,035 cm^−1^ and 1,160 cm^−1^ [41]. The divergence of band at around 1,035 cm^−1^ into two small peaks at 1,035 cm^−1^ and 1,045 cm^−1^ after pretreatment indicates removal of hemicellulose and accordingly increase of lignin. Regarding the bands at 1,160 cm^−1^, pretreated rachis shows stronger peaks than the untreated, while there are no obvious changes of peaks before and after pretreatment of leaflets, suggesting a more significant compositional change of cellulose and hemicellulose in pretreated rachis. The disappearance of band at around 1,247 cm^−1^ for pretreated leaflets is another evidence of the removal of hemicellulose by hydrothermal pretreatment. Considering changes to lignin fraction after pretreatment, peaks at 1,465, 1510, and 1,595 cm^−1^ are stronger than those before pretreatment, indicating the increase of lignin content after pretreatment due to the removal of hemicellulose.

### 3.4. Effect of Hydrothermal Pretreatment on Crystallinity Changes

Crystallinity in biomass is an important characteristic that has been shown to affect cellulose enzymatic digestibility [42]. Table 2 shows the crystallinity of leaflets and rachis before and after pretreatment based on Rietveld method. The powder patterns and Rietveld fits are included as Supplementary Material (Figure S.1 available online at http://dx.doi.org/10.1155/2015/216454). The set of refinement parameters used resulted in good fits (*R*_wp_ < 4.0%) of all samples, as well as realistic backgrounds, which is a prerequisite for accurate determination of crystallinity. The background level, in particular, highly depends on how the peak shape function is modelled. Testing different sets of refinable parameters, which all resulted in good fits, variations in crystallinity of about 2-3% were observed, which can be used as an estimate of uncertainty by this method, whereas the relative crystallinity between the different samples remained unaffected.

**Figure 4:**
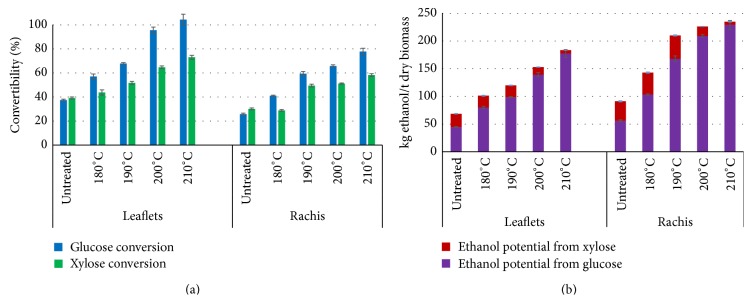
Glucose and xylose conversion and theoretical ethanol yield of untreated and treated date palm leaflets and rachis based on enzymatic hydrolysis. (a) Glucose and xylose conversion of untreated and pretreated leaflets and rachis by enzymatic hydrolysis. (b) Bioethanol potential derived from glucose and xylose of untreated and treated date palm leaflets and rachis based on glucose and xylose yield in enzymatic hydrolysis. Enzymatic hydrolysis was conducted at 50°C for 72 h with Cellic CTec2 (15 FPU/g DM) and Cellic HTec2 (1/9 weight of Cellic CTec2).

In general, the crystallinity in rachis is higher than in leaflets, and after pretreatment crystallinities of both leaflets and rachis increase. The crystallinity of pretreated leaflets gradually reduces from 43.2% at 180°C to 38.4% at 200°C. Similar downward trend of crystallinity is identified in pretreated rachis, decreasing from 47.3% to 40.4% at 180 and 210°C, respectively. The increase of crystallinity of leaflets and rachis after pretreatment is due to the removal of amorphous cellulose as well as xylan that is also amorphous [1, 42]. There is an obvious downside trend of crystallinity in pretreated biomass obtained by increasing temperature (180–210°C), showing the positive effect of pretreatment severity on reducing biomass crystallinity. Moreover, the reduction of crystallinity index during pretreatment could enhance cellulose digestion due to greater enzyme adsorption [43]. Consistent with this, higher cellulose digestibility of pretreated biomass was observed with lower crystallinity index (see Figure 4(a) and Table 2).

### 3.5. Enzymatic Digestibility of Solids from Pretreated Leaflets and Rachis

The enzymatic digestibility of solids from pretreated biomass is one of the most important indexes to evaluate the effectiveness of pretreatment. In this study, the advanced commercial cellulolytic enzymes, Cellic CTec2 and Cellic HTec2, were used in enzymatic hydrolysis of the untreated and hydrothermal pretreated biomass. The hydrothermal pretreatment was capable of enhancing enzymatic digestibilities of both leaflets and rachis (Figure 4(a)). There was a 60% enhancement of glucan-to-glucose conversion by pretreatment at 210°C/10 min (100% conversion) compared with untreated leaflets (40% conversion). Although the increase of xylan-to-xylose conversion was less than glucan-to-glucose conversion, it was still significant with about 35% increase. As for rachis, the pretreatment enhancements of glucan-to-glucose (52% increase) and xylan-to-xylose conversion (28% increase) were lower than the case for pretreated leaflets. Combined severity factor was also applied as an indicator of biomass digestibility in enzymatic hydrolysis. The coefficient of determination (*R* ^2^) between combined severity factor and glucose conversion of pretreated leaflets and rachis was 0.96 and 0.97, respectively. Theoretical ethanol yield was computed based on the glucose and xylose yield in enzymatic hydrolysis (Figure 4(b)). The highest theoretical ethanol yield was observed at 210°C/10 min for both leaflets (183.6 kg/t dry biomass) and rachis (235.0 kg/t dry biomass). As pretreatment temperature of leaflets increased, ethanol from glucose increased from 44.7 (untreated) to 177.7 kg/t dry biomass (pretreated at 210°C/10 min), while ethanol from xylose decreased from 24.0 (untreated) to 5.9 kg/t dry biomass (pretreated at 210°C/10 min). Similar trend was observed in case of rachis, where glucose-derived ethanol increased from 56.0 (untreated) to 229.3 kg/t dry biomass (pretreated at 210°C/10 min), while xylose-derived ethanol decreases from 35.2 (untreated) to 5.8 kg/t dry biomass (pretreated at 210°C/10 min).

The results show the great potential of applying hydrothermal pretreatment on processing date palm residues to produce bioethanol due to the boosts of enzymatic digestibility, which was also observed in other lignocellulosic biomass treated by hydrothermal pretreatment [14–17]. In general, the combined severity factor is applied for elaborating the removal of lignin and solubility of xylan that are two influencing factors of enzymatic hydrolysis. From this perspective, combined severity factor can be used to analyze enzymatic digestibility of pretreated biomass. In this study, very strong correlations between combined severity factor and glucan-to-glucose conversion were observed on both pretreated biomass, which could be a good indicator of enzymatic digestibility efficiency. Nevertheless, according to Pedersen's review on relation between pH and pretreatment severity [25], no evident correlations were observed between enzymatic hydrolysis (glucan-to-glucose conversion) and combined severity factor for acid steam explosion, alkaline wet oxidation, and lime pretreatment. Instead, there was some correlation between the sugar hydrolysis yields (glucose and xylose) and the pretreatment pH, but no correlation with the pretreatment temperature (90–200°C) based on a quantitative comparison of published data for wheat straw pretreatment [25]. However, it was suggested that temperature showed a greater influence on the pretreatment efficiency than predicted by the severity factor in single hydrothermal pretreatment of mixed hardwoods [44]. The conflicting conclusions were probably due to the different pretreatment methods and type of feedstock applied in previous studies. Combined severity factor was mainly determined by temperature in our study, making it reasonable that there are high correlations between combined severity factors and hydrolysis. In spite of that, it is necessary to expand the spectrum of conditions to get a validated and accurate index or model to reflect enzymatic digestibility for the sake of optimization of pretreatment. Bioethanol potential of biomass depends on glucose and xylose released in enzymatic hydrolysis (see (6)). Since there are no big changes of glucan recovery in fibers of pretreated biomass (see Figure 1(b)), the increasing contribution to ethanol by glucose is mainly due to the improvement of enzymatic convertibility of glucan-to-glucose. While the decreasing contribution to ethanol by xylose is because of the rapid decrease of xylan recovery along with increase of pretreatment temperature (Figure 1(c)), the optimal condition to maximize bioethanol potential for leaflets and rachis was found 210°C/10 min, where 183.6 and 235.0 kg ethanol/t dry biomass was available, respectively.

### 3.6. Fermentability Test of Pretreatment Liquids

**Figure 5:**
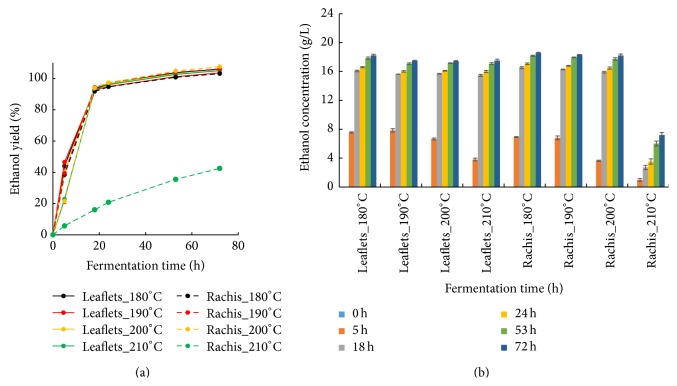
Ethanol yield and corresponding ethanol concentration in the fermentation of liquids from pretreated leaflets and rachis. (a) Ethanol yield in the fermentation of liquids from pretreated leaflets and rachis. (b) Ethanol concentration in the fermentation of liquids from pretreated leaflets and rachis. The fermentation was carried out at 32°C for 72 h with the yeast (*S. cerevisiae*) inoculation of 2 g/L.

In the pretreatment process, high portion of xylan and small amount of glucan and lignin were removed and solubilized into the liquid stream. As the pretreatment severity increased, a variety of degradation substances (organic acids, phenols, and furans) were generated. These compounds can be inhibitory for ethanol production [45]. In order to evaluate the inhibition to ethanol production, fermentability tests of pretreatment liquids from pretreated leaflets and rachis were conducted. Although strong inhibition was observed in liquids of rachis pretreated at 210°C, there were no inhibitions when the temperature was below 200°C (Figures 5(a) and 5(b)). After 18 h the ethanol yield and concentration values reached the same level for all the runs except for rachis treated at 210°C (Figures 5(a) and 5(b)).

The organic weak acids (formic acid and acetic acid) are the main fermentation inhibitors of ethanol production when comparing organic weak acids, phenols, and furans from steam exploded corn stover [46]. The critical inhibition concentration for acetic acid, furfural, and 5-(hydroxymethyl)furfural (HMF) is 6, >4, and >4 g/L, respectively [46]. The high concentration of acetic acid (18.28 g/L) rather than furfural (1.53 g/L) and HMF (2.15 g/L) in liquids from pretreated rachis at 210°C is speculated to be one of the factors responsible for the strong inhibition. The liquid stream after the pretreatment usually contains high amount of pentose especially xylose that cannot be metabolized by Baker's yeast (*S. cerevisiae*). However, the issue can be overcome by metabolic engineered yeast that is capable of cofermentation of pentose and hexose in both academia and industry [47], indicating high application potential of pretreatment liquids in the future.

### 3.7. Simultaneous Saccharification and Fermentation (SSF) of Pretreated Solids

**Figure 6:**
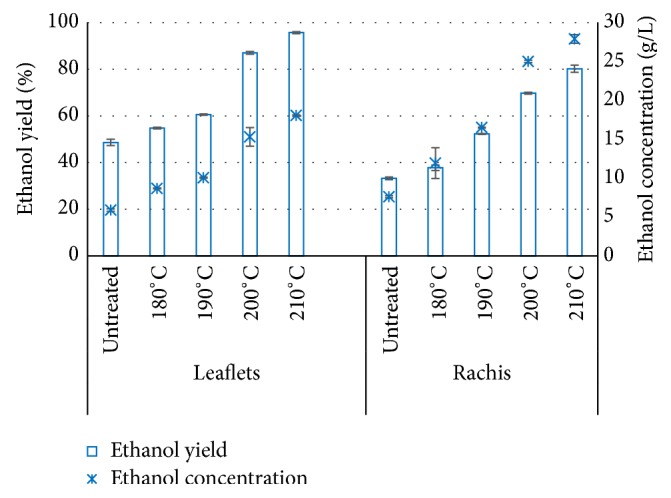
Ethanol yield and corresponding ethanol concentration in SSF of solids from untreated and pretreated date palm leaflets and rachis. Enzymatic hydrolysis (10% DM) was conducted at 50°C for 72 h with Cellic CTec2 (15 FPU/g DM) and Cellic HTec2 (1/9 weight of Cellic CTec2). In SSF, enzymatic hydrolysis was conducted for 24 h prior to fermentation that was carried out at 32°C for 72 h with the yeast inoculation (*S. cerevisiae*) of 2 g/L.

Simultaneous saccharification and fermentation was applied to investigate the effect of hydrothermal pretreatment on ethanol production (Figure 6). Ethanol yield of leaflets increases from 49% (5.89 g/L) to 96% (18.05 g/L) after pretreatment at 210°C/10 min. As for the enhancement of ethanol yield by pretreatment of rachis, it rose from 33% (7.59 g/L) to 80% (27.90 g/L). Higher ethanol yield was achieved in leaflets and rachis pretreated at higher temperature.

The results show that higher pretreatment temperature is favorable for higher ethanol conversion for both leaflets and rachis, which is in accordance with the trend in enzymatic hydrolysis. Low or even no inhibition is supposed to be in solids fraction after separation with liquids because fermentability test of pretreatment liquids shows almost no inhibition except for rachis treated at 210°C/10 min. In this case the hydrolysis is speculated to be the limiting step of SSF for production of ethanol. The comparable or even higher ethanol conversion (can be equivalent to glucose conversion) by SSF compared with glucose conversion in enzymatic hydrolysis shows that SSF seems to be a better method for ethanol production from date palm leaflets and rachis than separate hydrolysis and fermentation (SHF). The principal benefits of performing the enzymatic hydrolysis together with fermentation, instead of performing separate step after the hydrolysis, are the reduced end-product inhibition of enzymatic hydrolysis and the reduced investment cost. The principal drawbacks, on the other hand, are the need to find favorable conditions (like temperature and pH) for both enzymatic hydrolysis and fermentation [48, 49]. Optimization of SSF process is affected by several factors, such as cellulase concentration, substrate concentration, temperature, yeast loading, and incubation time [50–53]. The effect of these factors on ethanol production efficiency is planned to be a next step in our research in similar fashion to studies performed by Hari Krishna and Chowdary [50]. For the sake of tech-economic evaluation of the ethanol production from date palm residues, optimization of SSF process is also required in our future work.

## 4. Conclusions

Date palm is playing crucial role in the agriculture in the hot and arid regions. However, utilization of lignocellulosic residues from date palm to produce renewable bioenergy has not been well investigated. In this study, leaflets and rachis were treated by hydrothermal pretreatment. Physicochemical characterization, enzymatic hydrolysis, fermentability test, and SSF of pretreated biomass were applied to evaluate the effectiveness of hydrothermal pretreatment. Noticeable advantages were observed on facilitating structural deconstruction, achieving high sugar recovery, generating no significant fermentation inhibition, and enhancing enzymatic digestibility and ethanol conversion. Optimal pretreatment condition was observed at 210°C/10 min by achieving the highest bioethanol potentials.

## Supplementary Material

X-ray powder diffraction patterns and Rietveld fits of date palm leaflets and rachis before and after hydrothermal pretreatment.

## Figures and Tables

**Table 1:** FTIR absorbance bands in biomass study (adapted from [41, 54–56]).

Wavenumber (cm^−1^)		Polymer
900	Glycosidic bond	Cellulose (amorphous) [54]
1035	C-O, C=C, and C-C-O stretching	Cellulose, hemicellulose, and lignin [55]
1045	C-C, C-OH, C-H ring, and side group vibrations	Lignin [56]
1098	Glycosidic bond	Cellulose (crystalline) [54]
1160	C-O-C asymmetrical stretching	Cellulose, hemicellulose [55]
1247	C-O stretching	Hemicellulose, lignin [41]
1465	C-H deformation	Lignin [54]
1510	Aromatic ring vibration	Lignin [54]
1595	Aromatic ring vibration + C=O stretching	Lignin [54]
2840, 2937	C-H stretching	Lignin [54]
3421	O-H stretching	Lignin [54]

**Table 2:** Crystallinity analysis of leaflets and rachis before and after hydrothermal pretreatment according to Rietveld method.

	Samples				
	Leaflets	Leaflets_180	Leaflets_190	Leaflets_200	Leaflets_210
Crystallinity (%)	29.3	43.2	42.1	38.4	39.1
*R*_wp_ (%)^a^	3.80	3.57	3.56	3.73	3.78

	Rachis	Rachis_180	Rachis_190	Rachis_200	Rachis_210

Crystallinity (%)	39.5	47.3	46.2	45.4	40.4
*R*_wp_ (%)^a^	3.51	3.69	3.55	3.68	3.93

^a^The weighted profile residual is given as *R*_wp_ = [Σ_*i*_ *w*_*i*_(*I*_obs*i*_ − *I*_calc*i*_)^2^/Σ_*i*_ *w*_*i*_(*I*_obs*i*_)^2^]^1/2^, where *I*_obs_ and *I*_calc_ are the observed and calculated intensity, respectively, and *w* is the weighting function, 1/*I*_obs_.
