# Supplementary figures and images for: Hydrothermal Pretreatment of Date Palm (Phoenix dactylifera L.) Leaflets and Rachis to Enhance Enzymatic Digestibility and Bioethanol Potential
Source: Biomed Res Int. 2015 Aug 12;2015:216454. doi: 10.1155/2015/216454 (PMC4549489; doi:10.1155/2015/216454)

Leaflets

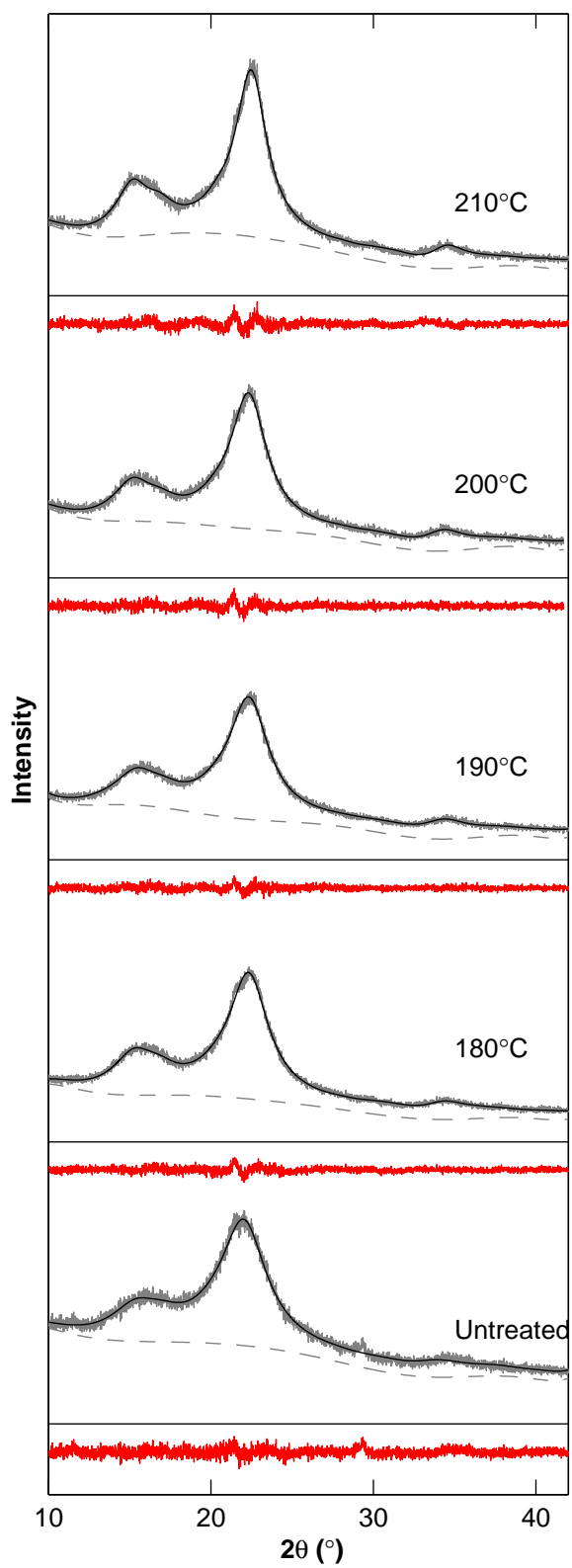

Rachis

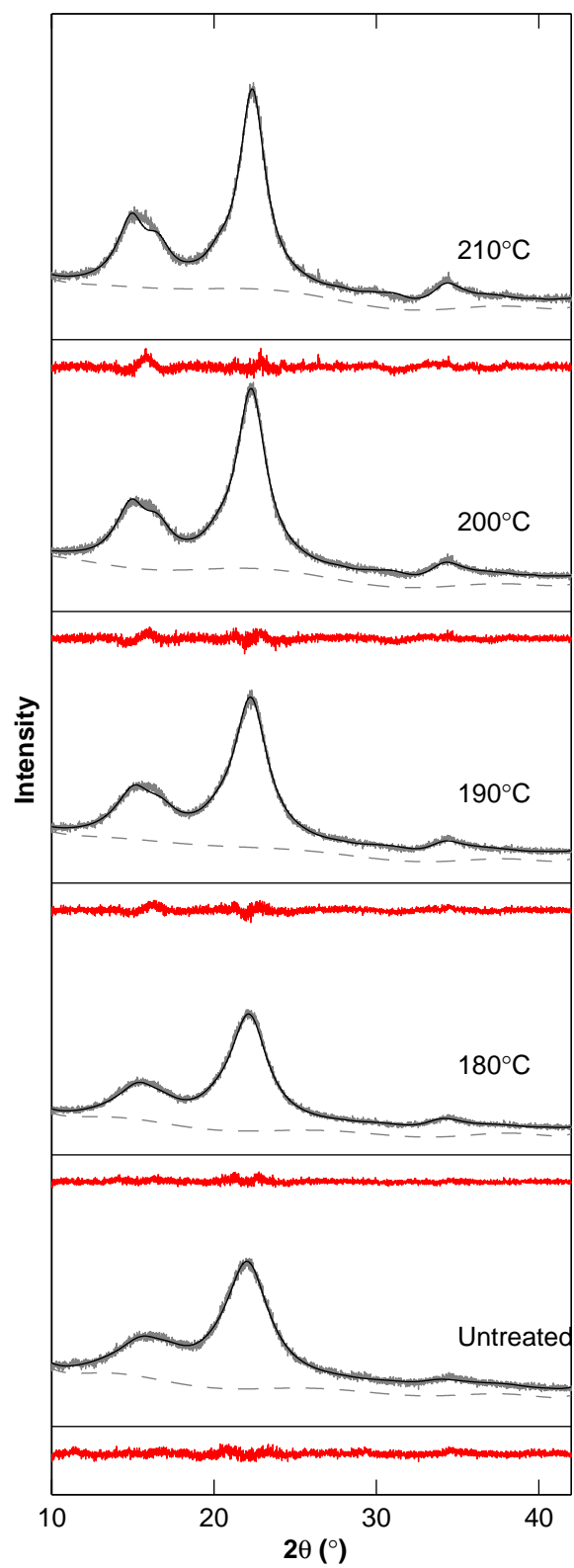

Supplement: Supplementary file 1 — X-ray powder diffraction patterns and Rietveld fits of date palm leaflets and rachis before and after hydrothermal pretreatment. [file 216454.f1.pdf]
